# Navigating Motivation: A Semantic and Subjective Atlas of 7 Motives

**DOI:** 10.3389/fpsyg.2020.568064

**Published:** 2021-01-27

**Authors:** Gabriele Chierchia, Marisa Przyrembel, Franca Parianen Lesemann, Steven Bosworth, Dennis Snower, Tania Singer

**Affiliations:** ^1^Department of Psychology, University of Cambridge, Cambridge, United Kingdom; ^2^Akkon University of Applied Sciences for Human Sciences, Berlin, Germany; ^3^Helmholtz Institute, Utrecht University, Utrecht, Netherlands; ^4^University of Reading, Reading, United Kingdom; ^5^Department of Economics, Hertie School of Governance, Berlin, Germany; ^6^Blavatnik School of Government, Oxford, United Kingdom; ^7^Social Neuroscience Lab, Max Planck Society, Berlin, Germany

**Keywords:** affiliation, care, motives, motivation psychology, semantic categorization, economic decision making

## Abstract

Research from psychology, neurobiology and behavioral economics indicates that a binary view of motivation, based on approach and avoidance, may be too reductive. Instead, a literature review suggests that at least seven distinct motives are likely to affect human decisions: “consumption/resource seeking,” “care,” “affiliation,” “achievement,” “status-power,” “threat approach” (or anger), and “threat avoidance” (or fear). To explore the conceptual distinctness and relatedness of these motives, we conducted a semantic categorization task. Here, participants were to assign provided words to one of the motives. By applying principal component analysis to the categorization assignments we represent the semantic inter-relations of these motives on a two-dimensional space, a “semantic atlas.” This atlas suggests that, while care and affiliation are conceptually close, affiliation is closer to threat avoidance (or fear); opposite to these motives we find achievement, consumption and power, with the latter lying closer to threat approach (or anger). In a second study, we asked participants to rate how well the motive-specific words obtained in the first study described their currently experienced feelings. We find that semantically close motives are also more likely to be experienced together, that is, we replicate most of the semantic relations in the “subjective atlas.” We discuss our findings in comparison to other multi-dimensional models of motivation, which show clear similarities. In addition to these motivational atlases, we provide a database of motive-specific words, together with the valence and arousal scores. These can be used for future research on the influence of motives on decision making.

## Introduction

Standard economic theory is typically agnostic with regards to the types of goals that agents generally pursue and assumes that agents are systematically driven only by stable preferences (Samuelson, 1938; Stigler and Becker, 1977). In contrast, decades of psychological research have suggested that many decisions are driven by particular motives, such as the motivation for achievement, power or affiliation (Thorndike, 1898; McDougall, 1932; Lewin, 1935; McClelland et al., 1953; Mowrer, 1960; Deci and Moller, 2005; Heckhausen and Heckhausen, 2010). In line with this, natural languages present a highly rich and structured vocabulary of motive-related words (Talevich et al., 2017). These motives are typically conceived both as trait-related dispositions and as context-sensitive states that lead subjects to experience particular types of incentives as pleasurable and rewarding, to strive for certain types of goals, and hence to activate particular behavioral tendencies and related decisions (Atkinson, 1964; McClelland, 1965; Emmons and McAdams, 1991; Pang, 2010; Rheinberg and Engeser, 2010; Schultheiss and Strasser, 2012). Similarly, recent advances in neurobiology have also begun to move beyond classic approach/avoidance interpretations of motivation, which have been prevalent in the field of biology, enriching it with a more motive-related vocabulary such as power-status (e.g., Eisenegger et al., 2011; Terburg and van Honk, 2013), affiliation (e.g., Feldman, 2012) and care (e.g., Alcaro and Panksepp, 2011; Valk et al., 2017). Finally, behavioral game theorists (e.g., Camerer, 2003), social dilemma researchers (Dawes, 1980; Kollock, 1998; Fehr and Fischbacher, 2003; van Lange et al., 2013) and neuroeconomists (Glimcher et al., 2013) have also documented that humans often pursue ends other than the maximization of their own payoff in economic interactions (Frank, 1988; Loewenstein and O'Donoghue, 2004), and have, for example, incorporated status-related or altruistic preferences in utility functions to characterize such behaviors (e.g., Loewenstein et al., 1989; Robson, 2001; Sally, 2001; Fehr and Fischbacher, 2003; Fehr et al., 2013).

To lay the grounds of an integrated motive-based framework for decision making, we first reviewed the literature in the domains of motivation psychology, neurobiology as well as behavioral economics. This review is summarized in Table 1. Based on this review, we converged on a preliminary set of six motives that pertain to motivation psychology or neurobiology and that are likely relevant for economic decision making: achievement, power-status, affiliation, care, threat avoidance (anger), threat approach (fear). In addition, moving from economics toward psychology, consumption is proposed as an exploratory seventh motive in psychology to potentially parallel the benchmark form of utility typically used in economic research. Of these seven preliminary motives, power, affiliation and achievement are classic well-known motives in motivation psychology (Table 1), whereas power and affiliation have also more recently begun to be studied in neurobiological research. Fear (or threat avoidance) and anger (threat approach) are prominent in both motivation psychology and neurobiology, care is a term used mostly in neurobiology (Table 1).

**Table 1:** Motives across disciplines.

	**Psychology**	**Neurobiology**	**Economics**
Consumption/Resource-seeking	Foraging, ownership (McDougall, 1932) Hedonism (Schwartz and Boehnke, 2004)	Wanting/appetitive; desire—seeking; foraging (Berridge and Robinson, 1998; Depue and Collins, 1999; Ikemoto and Panksepp, 1999; Lea and Webley, 2006; Panksepp, 2006; Alcaro et al., 2007; de Waal, 2007; Schultz, 2007; Delgado et al., 2008; Berridge et al., 2010) Lust and play (Nelson and Panksepp, 1998; Panksepp, 2005; Alcaro and Panksepp, 2011)	Utility (Samuelson, 1938; Stigler and Becker, 1977) In experimental economic settings, a benchmark utility function is frequently assumed to depend only on monetary or material payoffs (Camerer, 2003) Speculative psychological and neurobiological correspondents of such a benchmark interpretation of utility are presented on the left side of this row
Care	Intimacy (McAdams, 1980) (Weinberger et al., 2010) Nurturance (Murray, 1938; Weinberger et al., 2010) Pro-social altruism (Heckhausen, 2000) Help (Heckhausen, 1989) Idealism and family (Reiss, 2004) Compassion (Weinberger et al., 2010) Alloic sympathy (Apter et al., 1998) Benevolence (Schwartz and Boehnke, 2004)	Care (Nelson and Panksepp, 1998; Panksepp, 2005; Alcaro and Panksepp, 2011) Maternal love/parental care (Bartels and Zeki, 2004; Strathearn et al., 2009) Loving-kindness and compassion (Klimecki et al., 2012, 2014; Weng et al., 2013; Engen and Singer, 2015; Bornemann et al., 2016; Hildebrandt et al., 2017; Valk et al., 2017) Trust (Bos et al., 2012; Boksem et al., 2013)	Altruism (Hamilton, 1964; Becker, 1974; Palfrey and Rosenthal, 1988; Andreoni, 1990; Bruce and Waldman, 1990; Sally, 2001; Fehr and Fischbacher, 2003) Generosity in GTPs and charitable donations (Harbaugh et al., 2007; Small and Lerner, 2008; Eimontaite et al., 2013; Polman and Kim, 2013; Böckler et al., 2016; Tusche et al., 2016) Trust in GTPs (Kosfeld et al., 2005; Baumgartner et al., 2008; Singer and Steinbeis, 2009; Böckler et al., 2016; Chierchia et al., 2017) Cooperation in GTPs (Batson and Moran, 1999; Batson and Ahmad, 2001; Fehr and Fischbacher, 2004; Small and Lerner, 2008; Eimontaite et al., 2013; Polman and Kim, 2013; Chierchia et al., 2017; Bartke et al., 2019) Decreased punishment in GTPs (Batson and Ahmad, 2001; Singer and Steinbeis, 2009; McCall et al., 2014; Kirk et al., 2016)
Affiliation	Need to be liked or the need to belong (Murray, 1938; McClelland et al., 1953; Jackson, 1974; Weiner, 1990; Baumeister and Leary, 1995; Heckhausen and Heckhausen, 2010) Autic sympathy (Apter et al., 1998) Conformity (Schwartz and Boehnke, 2004)	Social bonding (Carter, 1998; Insel and Young, 2001; Ross and Young, 2009; Gordon et al., 2011; Feldman, 2012; McCall and Singer, 2012; Bakermans-Kranenburg and van Ijzendoorn, 2013; Rilling, 2013) (Fear of) social rejection (Eisenberger et al., 2003; Eisenberger, 2012) Tend and befriend (Taylor, 2006) Norm compliance, social influence and peer influence (Spitzer et al., 2007; Klucharev et al., 2009; Chein et al., 2011; Zaki et al., 2011; Izuma and Adolphs, 2013; Ruff et al., 2013; van Hoorn et al., 2016)	Social identity influence economic outcomes (Akerlof and Kranton, 2000) Norm-based cooperation and punishment in GTPs (Fehr and Fischbacher, 2004; Carpenter and Matthews, 2009; Fehr and Schurtenberger, 2018) Ingroup cooperation and outgroup punishment in GTPs (Bernhard et al., 2006; Balliet et al., 2014; Yamagishi and Mifune, 2016) Increased generosity in GTPs in response to social evaluation cues (Hoffman et al., 1996; Takahashi et al., 2007; Ekström, 2012; von Dawans et al., 2012; Winking and Mizer, 2013) Conformism in GTPs and economic decision making (Shang and Croson, 2009; van Hoorn et al., 2016; Charness et al., 2019; Dimant, 2019)
Power—Status	Power/status-seeking (Weiner, 1990; Schwartz and Boehnke, 2004) Having impact (Fodor and Riordan, 1995; Schultheiss and Brunstein, 2010) Being strong and influencing others (McClelland et al., 1953; Reiss, 2004; Heckhausen and Heckhausen, 2010) Agency/Power (Diehl et al., 2004; Abele et al., 2008)	Competition/dominance (Wingfield et al., 1990; Mazur and Booth, 1998; Salvador, 2005; Burnham, 2007; Eisenegger et al., 2010, 2011; Hall et al., 2010; Stanton et al., 2011; Apicella et al., 2015; Reimers and Diekhof, 2015; Dreher et al., 2016; Carré and Archer, 2018; Bird et al., 2019)	Status and power concerns (Frank, 1985; Cole et al., 1992; Robson and Samuelson, 2011) Punishment in GTPs (Straub and Murnighan, 1995; Yamagishi et al., 2012; Gordon and Lea, 2016; Chierchia et al., 2017) Status/image-based cooperation and generosity or withdrawal from cooperation in GTPs (Wedekind and Milinski, 2000; Hardy and Van Vugt, 2006; Lammers et al., 2008; Kumru and Vesterlund, 2010; Guinote et al., 2015) Risk taking (Anderson and Galinsky, 2006; Apicella et al., 2015) Impatience in temporal discounting (Joshi and Fast, 2013)
Achievement	Achievement/being better or more efficient than before (McClelland et al., 1953; Atkinson and Feather, 1966; Weiner, 1990; Schwartz and Boehnke, 2004; Heckhausen and Heckhausen, 2010)	Neurobiological research on achievement-related behaviors remains sparse	Pursuing subjective goals and striving for success (Kahneman and Tversky, 1979; Selten, 1998; Gilboa and Schmeidler, 2001; Gómez-Miñambres, 2012)
	Agency/Achievement (Diehl et al., 2004; Abele et al., 2008) Competence (Fiske et al., 2007; Cuddy et al., 2008) Mastery (Apter et al., 1998)		
Fear/Threat avoidance	Fear/Threat avoidance (Epstein, 1972; Heckhausen, 1989; Carver and Harmon-Jones, 2009) Harm avoidance (Murray, 1938) Avoidance (Thorndike, 1898; Lewin, 1935; Hull, 1943) Anxiety (Trudewind, 2000) Security (Schwartz and Boehnke, 2004)	Fear/Threat avoidance (Nelson and Trainor, 2007; Siever, 2008; Rodrigues et al., 2009; Bond and Wingrove, 2010; Potegal and Stemmler, 2010; Adolphs, 2013; Barrett and Russell, 2014) Fear/panic (Nelson and Panksepp, 1998; Panksepp, 2005, 2006; Alcaro and Panksepp, 2011) Punishment/reinforcement learning (O'Doherty et al., 2001; Dayan and Balleine, 2002; Montague et al., 2004; Schultz, 2007; Seymour et al., 2007; Rangel et al., 2008; Seymour and Dolan, 2008; Bromberg-Martin et al., 2010; Lang and Bradley, 2010; Dayan and Berridge, 2014)	Risk aversion, loss aversion and ambiguity aversion (Raghunathan and Pham, 1999; Lerner and Keltner, 2001; Maner and Gerend, 2007; Porcelli and Delgado, 2009; de Martino et al., 2010; Hartley and Phelps, 2012; Canessa et al., 2013; Schulreich et al., 2016)
Anger/Threat approach	Anger/Threat approach (Carver and Harmon-Jones, 2009) Aggression (Heckhausen, 1989) Rage (McDougall, 1932) Vengeance (Reiss, 2004)	Anger/Threat approach (Nelson and Trainor, 2007; Siever, 2008; Rodrigues et al., 2009; Bond and Wingrove, 2010; Potegal and Stemmler, 2010; Adolphs, 2013; Barrett and Russell, 2014) Rage (Nelson and Panksepp, 1998; Panksepp, 2005, 2006; Alcaro and Panksepp, 2011)	Punishment in GTPs (Pillutla and Murnighan, 1996; Andrade and Ariely, 2009; Seip et al., 2014; Gummerum et al., 2016; Liu et al., 2016) Withdrawal from cooperation in GTPs (Dunn and Schweitzer, 2005; Polman and Kim, 2013; Motro et al., 2016) Risk taking (Raghunathan and Pham, 1999; Lerner and Keltner, 2001; Fessler et al., 2004; Tsai and Young, 2010; Kugler et al., 2012; Ferrer et al., 2017; She et al., 2017)

*GTPs, game theoretical paradigms*.

Naturally, the short review summarized in Table 1 is far from exhaustive and the list of seven preliminary motives is unlikely conclusive either. Yet, these motives have been identified to be relevant for the three fields of interest reviewed here and should provide sufficiently important and diverse stepping-stones from which in future research on other related motives could be investigated. Rather than justifying the selection of these particular set of motives, the aim of this work is 2-fold: first, we aim to investigate how these seven relevant motives are related to each other—which of these motives are conceptually and/or experientially similar or different to each other? Which emerge together and which are rather antagonistic to each other? Second, we aim at providing a database of motive-specific words that may be used in future studies to more specifically probe the role of distinct motives in economic decision making.

To achieve these two goals we conducted two studies. First, we follow seminal efforts in the emotion literature (e.g., Russell, 1980) and investigate the semantic relations of the seven identified motives. We do so by asking whether and how subjects coherently differentiate between these motives in a semantic categorization task. By applying principle component analysis to such categorization judgments, we then further explore which dimensions might allow them to do so. In a second study, we assess whether the observed semantic inter-relations hold at the level of subjective experience, that is, whether semantically “close” motives are also more likely to co-occur in subjectively reported experience. In what follows, we begin by briefly introducing the seven motives of interests. We then pass to describing the two empirical studies and conclude by providing a database of motive-specific words.

### Motives in Motivation Psychology

Three motives, namely, “achievement,” “power,” and “affiliation” are among the most recognized motives in motivation psychology (Weiner, 1990). *Achievement* has been defined as the desire to do something better or more efficiently than before (McClelland et al., 1953; Atkinson and Feather, 1966; Weiner, 1990; Heckhausen and Heckhausen, 2010) or as “the need to feel that one is making progress toward important and/or long term goals” (Apter et al., 1998, p. 9). A *power* and/or *status-seeking* motive can be understood as the desire to have an impact (Fodor and Riordan, 1995; Schultheiss and Brunstein, 2010), to be strong, to influence others (McClelland et al., 1953; Reiss, 2004; Heckhausen and Heckhausen, 2010). It has also been described as “the need to be in control of objects, situations and events, to dominate people” (in Apter's “autic mastery,” Apter et al., 1998, p. 9). *Affiliation* has been defined as the need to be liked or the need to belong (Murray, 1938; McClelland et al., 1953; Jackson, 1974; Baumeister and Leary, 1995; Heckhausen and Heckhausen, 2010), and many have distinguished this from a *Care* related motive, which is rather related to accepting and nourishing others. For instance, Murray (1938) contrasts nurturance and affiliation, McAdams (1980) distinguishes an intimacy motivation from affiliation, Heckhausen (2000) discriminates affiliation vs. altruism/help, Jackson (1974) distinguishes between affiliation and nurturance, Apter et al. (1998) differentiates between autic sympathy (“the need to be admired, to be attractive to others, popular or loved,” p. 9) and alloic sympathy (“the need to care for, nurture, give to others, to enjoy the pleasure others receive from this,” p. 9), and Schwartz's system of universal values distinguishes benevolence and universalism from conformity (Schwartz and Boehnke, 2004).

In addition to classical motives such as achievement, power and affiliation discussed in motivation psychology, many have advocated a motivational threat system encompassing fear and anger. For example, fear-related motives have been described as harm avoidance (Murray, 1938), avoidance (Thorndike, 1898; Lewin, 1935; Hull, 1943), fear (H. Heckhausen, 1989), anxiety (Trudewind, 2000), security (Schwartz and Boehnke, 2004), while anger-related motives have been characterized as aggression (Heckhausen, 1989), negativism (Apter et al., 1998), rage (McDougall, 1932; Panksepp, 2006), and vengeance (Reiss, 2004). Naturally, fear and anger also bare the names of primary emotions, though emotion researchers have frequently highlighted their motivational components (Roseman, 2011, calls them “emotivations”) and related them to “threat approach” and “threat avoidance” (Carver and Harmon-Jones, 2009). Finally, the optimization of one's own utility and welfare, which plays a prominent role in economics, is often operationalized as the self-interested accumulation of goods (e.g., as monetary payoff in behavioral economics settings). This has no clear correspondent in psychology (but see Kasser, 2006; Kasser et al., 2014). However, a possibly related motivational value called hedonism has been suggested by Schwartz and Boehnke (2004), and defined as “pleasure or sensuous gratification for oneself (pleasure, enjoying life, self-indulgent)” (p. 239). Moreover, a consumption motive has also been associated with terms such as wanting, desire-seeking, or foraging and ownership (McDougall, 1932), especially in the animal and neurobiological literature (Berridge and Robinson, 1998; Depue and Collins, 1999; Ikemoto and Panksepp, 1999; Lea and Webley, 2006; Panksepp, 2006; de Waal, 2007; Delgado et al., 2008; Berridge et al., 2010).

### A Semantic and Subjective Atlas of Seven Motives

As a first step toward investigating the relations between these motives, we conducted a semantic categorization task in which we asked subjects to ascribe a number of motive-related words to the seven motivational categories posited above. This approach draws from a tradition of psychological studies on emotions that assesses subjects' conceptualization of affective states (Kuppens et al., 2013, for a review). For instance, in a seminal paper, Russell (1980) asked subjects to categorize 28 emotion-related words to pre-defined emotional categories and found that 2 dimensions, namely valence and arousal, could predict most categorization judgments (see also Posner et al., 2005; Kuppens et al., 2013). In line with this, many psychologists (e.g., Shaver et al., 1987; Barrett, 2004) have capitalized on categorization agreements/disagreements to spatially represent “closeness” of emotion-related concepts on a single (multidimensional) space. To our knowledge, these approaches have frequently been applied to emotions, but not to the motives addressed here.

Importantly, Russell (1980) argues that the cognitive structure of emotions should reflect the structure of actually experienced emotions, but this needn't always be the case. For instance, compassion tends to be prototypically conceptualized as a positive emotion, though when experienced (e.g., when observing a suffering other) it can be accompanied by positive as well as negative affect (Condon and Feldman Barrett, 2013). We thus asked whether the conceptual-semantic proximity of motives could predict their experienced proximity (e.g., an increased likelihood of co-occurrence)? To answer this question, in a second study, we asked participants to rate how well the motives of interest described their currently (spontaneously) experienced motives and feelings.

In terms of the expected relations between these motives, achievement and power are likely to be related, as are affiliation and care. In fact, a long standing tradition places power and achievement-related constructs within an “agency” dimension of behavioral orientation (which refers to a person's striving to be independent, to control one's environment, and to assert, protect and expand one's self), sometimes with achievement-related states on one end of this hypothetical agency spectrum, and power-related states on the other (Bakan, 1966; Diehl et al., 2004; Abele et al., 2008). The same tradition incorporates care and affiliation-themed constructs within a “communion” dimension (which refers to a person's striving to be part of a community, to establish close relationships with others, and to subordinate individual needs to the common good) (Bakan, 1966; Camille et al., 2004; Abele et al., 2008) and has frequently illustrated a gradient within this dimension, spanning notions related to care, on one side, to those related to “lack of independence” (i.e., affiliation), on the other (Abele et al., 2008). Similarly to this agency/communion tradition, Apter et al. (1998) proposes the meta-motivational distinction between mastery, which encompasses elements of power and achievement, and sympathy, which includes affiliation and care-themed states. Furthermore, Schwartz's system of universal values also finds that conformity and benevolence are contiguous values, in that their pursuit is not mutually exclusive, as is the case for power and achievement (Schwartz and Boehnke, 2004).

Second, we expected that words attributed to affiliation would have more conceptual proximity to fear, as research on affiliation has made a link between the need to belong and fear of rejection, social pain and separation distress (Panksepp, 2006; Weinberger et al., 2010; Eisenberger, 2012). In contrast, we also expected words related to care to be relatively farther from fear, since research on empathic concern distinguishes between care-related motives and negatively valenced states such as empathic distress (Batson et al., 1983, 1987).

Third, we expected words related to threat approach (i.e., anger) and threat avoidance (i.e., fear) to be relatively close. In fact, both motives are typically associated with high arousal and negative affect, and both are related to perceived threats (Lerner et al., 2015). Moreover, as anger has been associated with feelings of agency (e.g., approach or fight-related responses) (Carver and Harmon-Jones, 2009), we expected it to be closer to power and achievement than threat avoidance (typically associated with avoidance or flight).

Finally, as consumption was thought of as a parallel to the self-interested motivation traditionally assumed in economics, we expected consumption-specific words to be relatively close to agentic motives such as achievement or power/status and distant from words related to care.

## Study 1

### Methods

#### Participants and Procedures

Seventy nine participants (40 males, mean age = 32.4, SD = 13) took part in the study over 10 experimental sessions. Sessions were conducted in the computer lab of the Department of Social Neuroscience at the Max Planck Institute in Leipzig, Germany. Participants took part in the tasks from individually shielded computer cubicles. Participants of sessions 1 through 6 (*n* = 38, 21 males, mean age = 41.3, SD = 18.2) took part in the categorization task only (see below), while participants of sessions 7 through 10 (*n* = 41, 20 males, mean age = 26.4, SD = 3.8) additionally took part in the valence and arousal ratings (after the categorization task). Each participant took part in only one session and sessions were scheduled based on participant availability over a 2-month period. Experimental protocols were approved by local ethics committee (of the Medical Faculty of the University of Leipzig, n. 090-15-090032015) and all participants provided written informed consent for the anonymized treatment of their data.

#### Word Pre-selection

To pre-select motive-related words to use in the categorization task, we drew from a previously existing “free-association” database (Nelson et al., 2004), which was created by requiring participants to name the “first word that came to mind that was meaningfully related or strongly associated to the presented cue word.” This database provides over 72.000 “cue-associate” pairs. To draw motive-related words from this database we proceeded in two steps. First, for each of our motives, we searched the database for one or two most closely related words. We called these “anchor words.” Second, we searched the database for adjectives that had been associated to the anchor words in either the 1st or 2nd degree. For example, for our care motive we selected among associates of the anchor word “care” (1st degree), as well as among the associates of these associates–2nd degree. For most of our motives, the identification of anchor words was trivial. Specifically, we took the word “care” as an anchor word for the care motive, the word “power” for power-status, “fear and fearful” for threat avoidance (i.e., fear), “anger/angry” for threat approach (i.e., anger) and “achieve/achievement” for achievement. Two of the posited motives, namely, “affiliation” and “consumption,” did not have obvious equivalents in the database. For affiliation we thus chose the anchor words “friend/friendly,” while for consumption we chose the anchor word “want.” After having obtained a preliminary set of motive-associated words, we expanded this set through online synonym vocabularies.

#### Semantic Categorization Task

The pre-selected words were then used in the main categorization task of interest. Here, subjects were required to ascribe each word to one of the seven motives posited by our framework. Visually, participants were shown a list of candidate motive-related words (in randomized order), on the left side of the screen, and seven labeled “boxes” on the right (see Supplementary Figure 1). All box labels began with the phrase “motivated by…” and ended with one of the following: “…care for others,” “…the desire to belong,” “…power,” “…achievement,” “…anger,” “…fear,” and “…the desire for possession and consumption”^1^. Subjects were then asked to drag and drop each word into one (and only one) of the boxes. A list of 174 words was used in sessions 1 through 6. After session 6, finding that participants quickly accustomed to the task and easily managed to complete it in the allotted time, we increased the number of words to increase our power. Specifically, participants of the remaining sessions (sessions 7 through 10) assessed a longer list of 304 words. With one exception (due to a programming error for the word “gemein” = mean), all of the words used in the first list were also used in the second. All participants assessed all of the words.

#### Valence, Arousal, and Frequency Scores

After the categorization task, participants of sessions 7 through 10 (*n* = 41) also rated each of the 304 words on two scales: a valence scale and an arousal scale. Scales consisted of visual analog scales, which could take values between 0 and 100 (in Supplementary Table 1 we subtract 50 from the mean valence and arousal scores, so that *t*-tests against 0 may suggest which words are statistically high/low in valence or arousal). Valence and arousal scales were altered relative to more commonly used ones in emotion research (e.g., scales from 1 to 9) (Bradley and Lang, 1999) to allow for greater variance in responses, for consistency with the self-report scales of study 2 (see below) and because we mainly intended to use such scales in a PCA that standardizes all measures to eliminate scale-specific variance. The instructions for the tasks were taken from Kanske and Kotz (2010). English translations in Supplementary Table 1 were carried out by professional translators.

#### Analysis

We ran two types of analyses. The first searched for “motive-specific” words, that is, words that were attributed more frequently to one motive than any other. To obtain these, for each word, we took the two categories in which the word had been most frequently placed in and we then ran a chi-square test to assess whether the resulting proportion could be due to chance. If this test was significant (*p* < 0.05), this indicates that assignment frequencies differed between the most popular and second-most popular categories (and thus also between the most popular category and the third-most popular category, the fourth-most popular etc.). We considered words surpassing this test to be “motive-specific,” because they were more frequently assigned to one category than any other, and we took words to be “ambiguous” otherwise. *P*-values were also corrected for multiple comparisons (using the Hochberg method). In Supplementary Table 1, we append an asterisk to the words that survived this more stringent criterion. This could be considered a statistical parallel to Russell's “precision” (which took a value of 0, if a given word was distributed randomly over the provided categories, and 1, if all subjects attributed a given word to the same category) (Russell, 1980). Second, we ran a principle component analysis (PCA) taking the seven motivation-categories as variables/descriptors, and the single words as items. The PCA was conducted with the PCA function of the “Factominer” package (Lê et al., 2008) in R (R-Core-Team, 2014). The PCA was based on the correlation matrix of the standardized variables and it used unrotated principle components. As supplementary variables of interest we took: (i) the results of the first analysis (namely, a vector indicating which motive—if any—each word had been specifically ascribed to); and (ii) valence and arousal scores. Supplementary variables enable to relate variables to PCA space, without affecting its solution (Lê et al., 2008).

### Results

#### Motive Specific Words

For each one of the tested motives, we found a subset of words that significantly (*p* < 0.05) differentiated this motive from each of the other presented motives. This was observed for 213 (70%) of the 304 words and an average of 30 words (SD = 8.28) per motivational category. This suggests that participants displayed on which words best characterize each of the seven distinctions we proposed. The “motive-specific” words are presented in the database (Supplementary Table 1). Within each motivational category they are ordered based on how frequently they were placed in that category.

#### Semantic Closeness of Seven Motives

**Figure 1:**
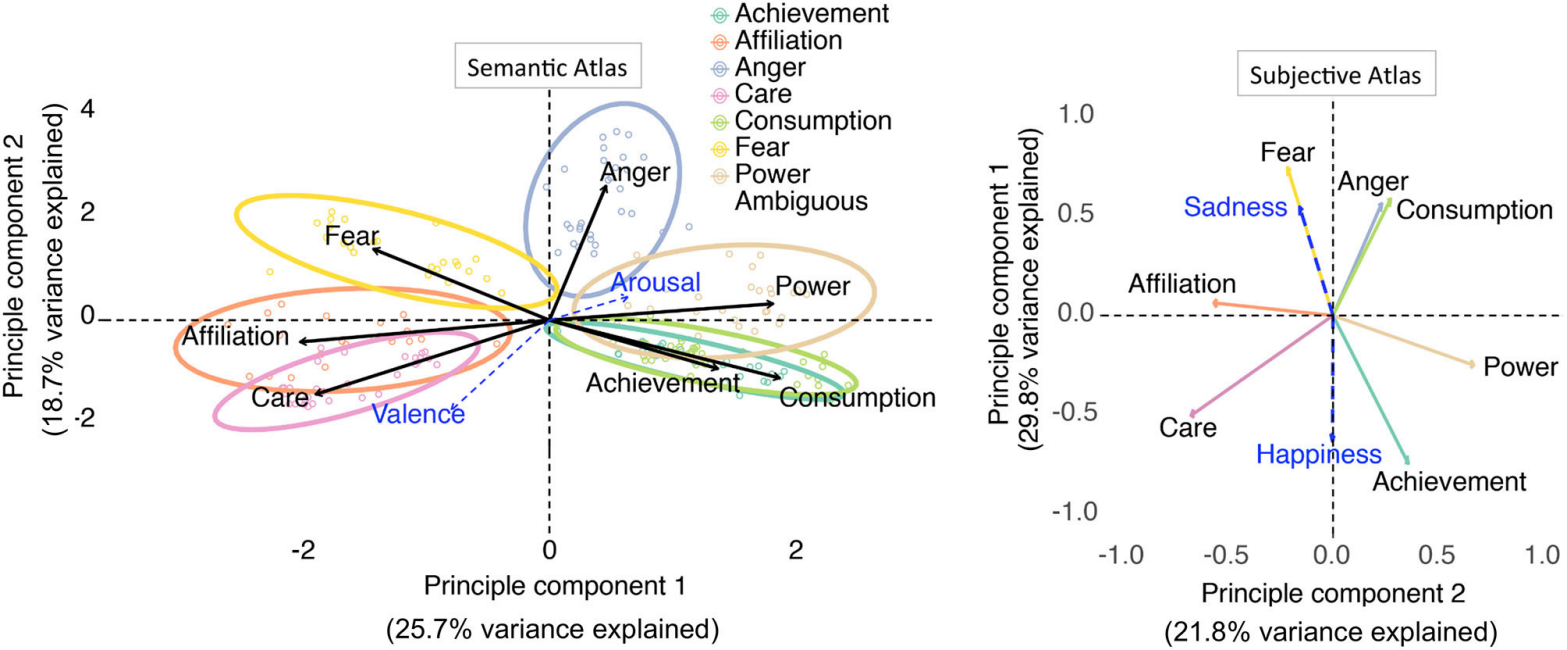
A semantic and subjective atlas of seven motives. **(Left)** *A semantic atlas of seven motives*. Principle component analysis (PCA) of a word categorization task in which participants were to assign words (points) to 1 of 7 motivational categories (arrows). Dots depict words more frequently attributed to one motivational category than any other (*p* < 0.05) are surrounded by 95% confidence ellipses. Words that did not have this property are labeled “ambiguous.” They were included in the PCA analysis but are not depicted (i.e., in white) to reduce visual clutter. Blue arrows represent how the valence and arousal of all words related to the PCA space. **(Right)** *A subjective atlas of seven motives*. Principle component analysis (PCA) of a self-report task in which participants were to rate on a visual analog scale how well a number of motive-specific words obtained in the first study described their spontaneously experienced motives and feelings at that moment.

To explore the relative “semantic distance” between motives we ran a PCA on the categorization judgments. The Kaiser rule suggested retaining the first 2 components (as these had eigenvalues higher than unity) of the PCA. The first component explained 25.7% of the categorization variance, the second 18.7%, for a total of 44.4%. The 1st component most clearly separated between affiliation, care and fear, on the one side, and power, achievement, and consumption on the other (all scores were below −0.3 and above 0.3, respectively). The 2nd component distinguished between anger and fear, on the one side, and care, consumption and achievement on the other (all scores above 0.4 and below −0.3, respectively) (see Figure 1, left). With regard to the supplementary valence and arousal variables, valence was correlated to both the PCA components, but more strongly to component 2 (*r* = 0.54, *p* < 0.001), while arousal was not clearly related to either of the dimensions (both r_s_ < 0.21).

## Study 2

### Methods

#### Participants

Participants (*n* = 310, mean age = 27.19, sd = 5.97, females = 163, males = 147) were recruited via email through the Max Planck participant database to take part in two separate studies on economic decision making (Chierchia et al., 2017, 2021). Those studied required a larger sample size than study 1, yet they provided a good opportunity to test these hypotheses as well. The higher sample size of study 2, is thus just a by-product of this incidental annexation. As soon as participants were welcomed to the experimental sessions, and thus before being introduced to any of the experimental activities of interest for those studies, they took part in the task described below. Assessments were approved by the Research Ethics Committee (agreement number 090-15-09032015) of the University of Leipzig, Germany. All participants provided written informed consent for the anonymized analysis of their data. We report all measures, manipulations, and exclusions in these studies, though no participants were excluded from the analysis.

#### Procedures

Participants were provided with a list of words and for each one, they were asked to rate in how far they felt “currently driven by these motivations, feelings or states,” on a continuous visual analog scale ranging −350 to 350 (numbers were not visible to participants). These novel questionnaire items probed the seven motives of interest and were taken from the motive-specific words individuated in study 1, above. Specifically, the fear-related items were “apprehensive,” “afraid,” “timid,” “nervous,” “panic-stricken,” “overcautious,” “frightened,” “reserved”; the anger-related items were “aggressive,” “angry,” “offended,” “irritable,” “argumentative,” “tempestuous,” “spirited,” care-related items were “caring,” “protective,” “kind-hearted,” “cordial,” “helpful,” “affectionate,” “sympathetic,” and “consoling”; power-related items were “mighty,” “dominant,” “authoritarian,” “firm,” “influential,” “condescending,” and “officious”; achievement items were “hard-working,” “industrious,” “capable,” “efficient,” “ambitious,” “success-driven,” “obstinate,” “productive”; affiliation-related items were “entertaining,” “ingratiating,” “excluded,” “conventional,” “attached,” “obliging,” “popular”; and consumption items were “consumerist,” “hoarding,” “avaricious,” “greedy,” “materialistic,” “cheap,” “pleasure-seeking,” “acquisitive,” “desire to buy,” “gluttonous.” In addition to these motive-related constructs, affect was measured by asking participants to provide ratings on a number of items related either to happiness (“content,” “happy,” “overjoyed,” “pleasant,” “enthusiastic”) or sadness (“sad,” “downcast”). Overall, the questionnaire consisted of 63 items. The order of the items was fully randomized for each participant, who viewed seven items per page.

#### Analysis

The ratings on items related to each motive displayed acceptable internal reliability: fear (Cronbach's α = 0.87), anger (α = 0.83), power (α = 0.86), care (α = 0.92), achievement (α = 0.88), affiliation (α = 0.67), consumption (α = 0.84). We thus first ipsatized the ratings for each subject and then aggregated over the items related to each motive. This resulted in 7 motive-related and 2 affect-related measures, for each participant. As for study 1, we then subjected these measures to principle component analysis, focusing on the seven motive-related measures as variables of interest and using the two affect measures as supplementary variables. As we aimed to establish whether the dimensions that explain the most variance at the semantic level (as investigated in study 1) also do so at the subjective level (as investigated here), we focused on the first two components of the PCA solution.

### Results

#### Subjective-Experiential Closeness of Seven Motives

Taken together, the first two components of a PCA solution explained 51.6% of the seven spontaneously occurring motives of interest, as measured by ratings on a visual analog scale (Figure 1, right). The first of the components (which explained 29.8% of the total variance) most clearly differentiated between fear, anger and consumption on the one hand (all scores > 0.56) and achievement and care on the other (scores < −0.50). The second component (which explained 21.8% of total variance) differentiated between more clearly between care and affiliation on the one end (both scores < −0.56), and power and achievement on the other (scores > 0.35). As for the supplementary variables, happiness and sadness were both (oppositely) related to the 1st dimension (with scores respectively >0.54 and < −0.63), with the exception of consumption, these dimensions clearly resembled those individuated in study 1, as can be observed in Figure 1 where, to facilitate comparability with the corresponding figure of study 1 (Figure 1, left), we plotted the 1st component (i.e., the component that explained the most variance) on the y-axis and the 2nd on the x-axis.

## Discussion

As a first step toward a motivation-based decision making framework that integrates psychology, neurobiology and behavioral economics, the main goal of the present paper was to investigate how seven relevant motives that have consistently recurred in one or all of these reviewed fields relate to one another, both on the semantic-conceptual as well as the subjective-experiential level. This preliminary set of motives are consumption/resource-seeking, care, affiliation, power-status, achievement, threat avoidance, and threat approach. The second goal of the present paper was to provide a word list of motives that could be used in future experimental studies on the influence of motives on economic decision making.

The analyses of a word-categorization task revealed that subjects coherently differentiated between the seven motives, as they agreed on which words were more related to one motive as compared to any of the others. In spite of this, participants disagreed more frequently on how to categorize words to some motives more than others. This enabled to obtain a spatial proxy of the relative conceptual overlap of each of the tested motives (Figure 1, left). The 1st dimension of the space differentiated between care, affiliation and fear on one side and power, achievement and consumption on the other. As anticipated, this separation is reminiscent of a communion/agency distinction (Bakan, 1966; Diehl et al., 2004; Abele et al., 2008), or a mastery/sympathy distinction (Apter, 1984; Apter et al., 1998), with care and affiliation words loading on one side, and power, achievement and consumption loading on the other. The 2nd dimension was partially related to valence and especially differentiated threat approach, threat avoidance, and power, on the one side, from care, affiliation, achievement and consumption, on the other. These inter-relations amongst motives were not only apparent in the semantic but also in the subjective atlas.

In both atlases, we found that care and affiliation were conceptualized and experienced as close but were differentiated along a previously reported valence related gradient (Abele et al., 2008) within the communion side of the space: most of the negatively valenced communion-related words were in fact attributed to the affiliation motive (e.g., “conventional,” “excluded,” “attached/clingy,” “ingratiating,” “conformist” etc.), while all but one care-specific word had positive valence (i.e., “pampering,” as opposed to “warm-hearted,” “soft-hearted,” “motherly,” “kind,” “understanding” etc.). In line with this, many positively valenced affiliation-specific words (e.g., “talkative,” “affable,” “gregarious,” “communicative,” “sociable”) did not survive correction for multiple comparisons, suggesting that, as affiliation-related words become positive, they also become more “conflated” with care. These results suggest that care and affiliation are partially distinguished by valence. From a more conceptual perspective we speculate that the distinction between care and affiliation could also be related to the fact that nearly all care-specific words clearly denoted other-oriented motivation and behaviors, a motive to protect/help/accept others (e.g., “selfless,” “generous,” “sacrificing,” “unselfish,” “helpful,” “protective” etc.) while those related to affiliation expressed a more self-focused need to be protected/helped/accepted or liked *by* others (e.g., “clingy,” “popular,” “attached”). This is in line with Apter's distinction between alloic vs. autic sympathy, and colleagues (1998), i.e., respectively, the motivation to nurture and love others vs. the need to be loved. Such a view is also compatible with the benevolence vs. conformity distinction in the quasi-circumplex model of Schwartz and Boehnke (2004), where benevolence is ascribed to the higher-order construct of “self-transcendence”– values which “promote the welfare of others, close and distant, and of nature” (p. 236)—while conformity is ascribed to the higher-order construct of “conservation”—values which “preserve the status quo and the certainty it provides (security, conformity, tradition)” (see Schwartz and Boehnke, 2004, p. 236). Notably, Schwartz and colleagues find that, despite this important distinction, conformity (i.e., affiliation in our model) and benevolence (i.e., care) are relatively proximal, as our data also suggests, despite noticeable methodological differences.

We also found that affiliation is semantically conceptualized and subjectively experienced as closer to threat avoidance (or fear) than care. Previous literature has frequently linked affiliation to various negatively valenced states of social anxiety, social exclusion or separation distress (Eisenberger et al., 2003; Eisenberger, 2012). Moreover, a separate line of studies on empathy for pain and altruistic behavior have shown that when distress prevails over empathic concern, subjects tend to make more self-protective and avoidant choices rather than altruistic ones (Batson et al., 1983, 1987; Singer and Klimecki, 2014). Our second finding could thus further qualify with our first one: affiliation-related concepts may be more negatively valenced than care-related concepts, partly because the motive of affiliation seems connected to the fear of separation and not belonging, and thus being more closely connected to the motive of fear. Similarly, in Schwartz and Boehnke's (2004) multi-dimensional model of values (Schwartz and Boehnke, 2004), security and conformity (analogs to our threat-avoidance and affiliation motives) are immediate neighbors, and both belong to the higher-order value of Conservation. In line with this, benevolence (similar to our care motive) is more distant from security values than conformity.

Moreover, and again similarly to Schwartz and Boehnke (2004), we found that power-status and achievement-related motives were also relatively close (as well as antagonistic to care and affiliation). Indeed, Schwartz and Boehnke (2004) propose that one higher-order value, self-enhancement [“to enhance (…) own personal interests even at the expense of others,” p. 236], combines power and achievement [and forms “a bipolar dimension with the higher-order type called self-transcendence, that combines universalism and benevolence values.” (p. 236)]. In line with this, power and achievement have also been previously indirectly associated with agency (Bakan, 1966; Diehl et al., 2004; Abele et al., 2008), which has been suggested to span from positive aspects of agency (such as “independence” and “achievement”), to more “excessive” forms of agency, such as “hunger for power and superiority,” culminating “in aggressive or rude behavior” (Bakan, 1966; Diehl et al., 2004; Abele et al., 2008). This agency spectrum appeared to be reflected in our motivational space as well, with power/status-related words (e.g., “influential,” “dominant,” “mighty,” “despotic” etc.) standing vertically between those related to more positively valenced achievement (“determined,” “success-driven,” “ambitious,” “motivated,” “capable,” “efficient,” “competitive” etc.) and those related to anger (“aggressive,” “hateful,” “hostile,” “argumentative” etc.). With regard to the content of the two sets of words, it also appears that achievement-specific words are more related to performance and seem only “incidentally” social. For instance, among the achievement-specific words, only the word “competitive” appears to imply some social comparison, while most power-related words more explicitly refer to dominance over others. Schematically, achievement could be conceptualized as socially neutral, or a-social, while power appears more frequently anti-social (van Honk et al., 2016).

Our fourth hypothesis, also substantiated in the results, was that threat approach and threat avoidance would be conceptually close to one another. The proximity of these two motives is in line with the notion that they can be considered as two opposing response tendencies (approach vs. avoidance) to perceived threats (Carver and Harmon-Jones, 2009). Indeed, anger and fear-related states are generally both low in valence and high in arousal, they are frequently experienced together (Moons et al., 2010), and they have been frequently found to cluster together in previous word sorting tasks (Russell, 1980; Posner et al., 2005; Kuppens et al., 2013). Interestingly, even though threat avoidance and threat approach both stem from an over-arching threat system and may thus be characterized as two sides of one coin, they can still be distinguished on the level of behavior, affective states, physiological arousal and underlying activation patterns (Stemmler, 2004; Stemmler et al., 2007). In line with this, we find that threat approach and avoidance display close spatial proximity, but are nonetheless distinct. The multi-dimensional model of human values (Schwartz and Boehnke, 2004) does not entail a motive or value similar to threat approach. However, in the motivational style profile (Apter et al., 1998), “negativism” (associated with acting provocatively and/or reacting angrily to situations) is treated as part of the meta-motivational oppositional modes of negativism vs. conformity. Such an opposition implies that the respective motivational modes are inherently antagonistic. Similarly, we find large spatial distance between anger and affiliation (see Figure 1).

The only aspect on which the semantic and subjective atlases diverged was with regard to our more exploratory consumption motive. In both atlases, consumption was related to our alleged “agency” dimension, however, in the semantic atlas, consumption overlapped highly with achievement and less with power, while in the subjective atlas it shifted away from achievement, and toward threat approach/anger. Though unanticipated, we speculate that this difference could be due to the fact that consumption-oriented behaviors are frequently conceptualized as linked to achievement (or perhaps even rationalized in the name of achievement). Similarly, in the theoretical model of relations among 10 motivational types of values (Schwartz and Boehnke, 2004), hedonism, which comes close to the consumption motive suggested here, can be found in close neighborhood to achievement. This proximity is in line with the notion that self-interested maximization of profit or money is frequently conceived as “an indicator of achievement, respect, and freedom or power” (Lea and Webley, 2006, p. 170). However, at the level of experience, it has also been suggested that the pursuit of profit (i.e., consumption) can be used as a socially acceptable or ritualized tool to express aggressive instincts (Behrendt, 2006), and that anger can be a “hidden motivator” behind the desire to buy products (Veling et al., 2011). Our finding that anger and consumption are subjectively experienced but not semantically conceptualized as close are in line with this notion, that anger and consumption may share a subconscious link (Behrendt, 2006; Veling et al., 2011). Furthermore, in the quasi-circumplex model of the 10 motivational types of values (Schwartz and Boehnke, 2004), hedonism is situated opposed to benevolence. Similarly, we find that care and consumption are spatially located on the opposite sides of the atlases.

One question our pattern of results may raise is that much literature has found communion and agency to be independent dimensions rather than negatively related dimensions (e.g., Abele et al., 2008). However, anti-correlated agency and communion dimensions have also been reported. For example, Abele et al. (2008) observe such an anti-correlation and suggest this may be due to the fact that they chose words in order to maximize the “separation” of the constructs of interest (agency and communion in their case). This also applies to our studies, and to study 2 in particular, given that the words chosen for the subjective-experiential ratings were those that maximally differentiated between motives in the categorization task (i.e., participants were likely to assign those words to one motivational category more than any other). Moreover, as again suggested by Abele and colleagues, “the finding that agency and communion are negatively related *if valence is controlled for* is in line with previous research” (p. 1214, cursive ours). Our valence-related PCA dimension might have captured valence-related variance, thus partially allowing such a negative correlation of agency and communion-related motives to surface. Finally, it should also be noted that, even though not prominent in the agency/communion literature, the antagonistic nature of care/affiliation vs. power/achievement is more central in the model of human values presented by Schwartz and Boehnke. Here in fact, the closest value correspondents of these motives (namely benevelonce/conformity vs. power/achievement) are assigned to opposite ends of an explicitly bipolar higher-order value construct, which separates between “Self-enhancement” (the motivation to enhance own personal interests, even at the expense of other) and “Self-transcendence” (the motivation to promote the welfare of others, close and distant, and of nature). Overall, this suggests that the negative relation of agency and communion-related motives can be reconciled with previous findings.

Finally, our sorting task also enabled to provide a list of 213 motive-specific words (Supplementary Table 1). Many of these words corroborate existing questionnaire items related to achievement, power, fear, anger and what we refer to as care (Lefcourt, 1991; Buss and Perry, 1992; Costa and McCrae, 1992; Lachman and Weaver, 1998; Abele et al., 2008; Spielberger's State, 2010). In addition, our findings also suggest that some words that have often been used in the literature may not be optimal to disambiguate between certain motives. For instance, the words “cordial” or “likable” are related to “warmth” in the NEO-PIR but also to affiliation tendency by Mehrabian's scale of Affiliative Tendency (Mehrabian, 1994). Our results indicate that, when confronted with the choice, participants indeed might associate these words with care rather than with affiliation. Researchers juxtaposing self-report measures to (economic) decision making paradigms (Maner et al., 2002; Bosman et al., 2005; Ben-Shakhar et al., 2007; Reuben and van Winden, 2008; Hopfensitz and Reuben, 2009; Eimontaite et al., 2013) could use our motive-specific words to better identify the motives underlying specific decisions and investigate whether indeed such motives are related to the corresponding decision patterns anticipated above (Table 1). In fact, recent studies have already adopted a subset of the motive specific words obtained here and found that they can be simultaneously sensitive to corresponding motivational inductions (e.g., of care, power, fear, or anger) and predictive of economic behaviors of interest (Chierchia et al., 2017, 2021; Bartke et al., 2019).

Future studies could use some of these words using spatial arrangement methods (Richie et al., 2020), in which no pre-existing categories are given and participants freely arrange words on a screen, based on their perceived similarities (such that words that are spatially closer are perceived to be more similar). This would allow to compute semantic distances based on a continuous measure rather than a categorical one. It would also allow investigating whether our results hold—that is, whether similar dimensions would differentiate between motives—when no over-arching categories are provided at all. This would provide further support to the notion that, much as in an atlas, there could be a useful underlying coordinate system to help navigate between important motives in economics, biology and psychology: a dimension that distinguishes between agency and communion on one side, and a positive and negative affect on the other.

## Conclusion

Integrating literature from motivation psychology, neurobiology and behavioral economics, we propose that at least seven distinct motives are likely to be crucial driving forces of human decision making: “consumption/resource seeking,” “care,” “affiliation,” “achievement,” “status-power,” “threat approach,” and “threat avoidance.” As a first preliminary investigation of such a motivational taxonomy, we analyzed how participants differentiate between these seven motives, and how such motives are inter-related on a semantic-conceptual as well as subjective-experiential level. We find that whereas care and affiliation are close, affiliation is closer to threat avoidance (or fear) and could thus refer to a more self-centered need to belong and to fear of social exclusion, whereas care is more positively valenced and could denote more altruistic orientation. On the other side of both semantic and experiential atlases, we find achievement overlapping highly with power, but with power lying closer to threat approach (or anger). Together with these atlases, we also provide a database of motive-specific words to be used in future research linking motives to decision making. Overall, we prospect a decision making model that goes beyond approach and avoidance, that is psycho-biologically plausible, and that highlights the importance of contexts in modulating motivation (e.g., Bosworth et al., 2016). Such a model could justify policies focusing on how institutions, contexts and frames can affect motives, and possibly harness them to bolster cooperation.

## Data Availability Statement

The raw data supporting the conclusions of this article will be made available by the authors, without undue reservation.

## Ethics Statement

The studies involving human participants were reviewed and approved by the local ethics committee of the Medical Faculty of the University of Leipzig (n. 090-15-090032015). The participants provided their written informed consent to participate in this study.

## Author Contributions

TS and DS conceived the study and together with GC and FPL, designed the study. MP provided the theoretical backbone by carrying out a comprehensive literature review on motivation psychology and biology and SB on economic theory. FPL and GC coordinated the empirical studies, collected the data, and carried out the statistical analyses. All authors hefcvm-07-626975 - Crx2lped draft the manuscript and gave final approval for publication.

## Conflict of Interest

The authors declare that the research was conducted in the absence of any commercial or financial relationships that could be construed as a potential conflict of interest.

## Footnotes

^1^For sessions 6 through 10, we slightly changed the label of this category to “motivated by wanting.” We did so to control for a possible overly negative connotation of the words consumption and possession. This label change led to no identifiable difference in the results.
