# Supplementary material for: Navigating Motivation: A Semantic and Subjective Atlas of 7 Motives
Source: Front Psychol. 2021 Jan 27;11:568064. doi: 10.3389/fpsyg.2020.568064 (PMC7874174; doi:10.3389/fpsyg.2020.568064)
Supplement: Supplementary file 1 [file Table_1.docx]

**Supplementary Table 1. Motive-specific words.**

Words that participants attributed to one motivational category significantly more than any other (uncorrected). Asterisks indicate additional Hochberg correction for multiple comparisons. In the valence and arousal columns, asterisks indicate whether values significantly differ from 0. PC1 and PC2 indicate scores on the first and second PCA components. *=p<0.05, **=p<0.01, ***=p<0.001.

| **word** | **motive** | **valence** | **arousal** | **PC1** | **PC2** |
| --- | --- | --- | --- | --- | --- |
| hard-working [leistungsbereit] | achievement*** | 8.55 (24.09) | 2.73 (27.4) | 1.76 | -1.42 |
| industrious [fleißig] | achievement*** | 25 (16.07)*** | 4.45 (24.52) | 0.76 | -0.81 |
| capable [leistungsfähig] | achievement*** | 24.6 (15.83)*** | 4.23 (26.29) | 1.63 | -1.32 |
| productive [produktiv] | achievement*** | 22.83 (15.57)*** | 1.77 (26.73) | 1.44 | -1.22 |
| overambitious [streberhaft] | achievement*** | -6.35 (22.68) | -11.75 (25.18) | 1.3 | -1.35 |
| ambitious [ehrgeizig] | achievement*** | 13.25 (18.6)** | 10.17 (22.12) | 1.66 | -1.35 |
| pressure-to-achieve [Leistungsdruck] | achievement*** | -19.43 (18.8)*** | 3.77 (30.8) | 0.83 | -0.6 |
| talent [Talent] | achievement*** | 31.35 (14.35)*** | 3.1 (30.85) | 0.81 | -0.77 |
| success-driven [erfolgsorientiert] | achievement*** | 9.38 (22.74) | 2.95 (26.69) | 1.7 | -1.19 |
| self [selbst-optimierend] | achievement*** | 15.42 (22.18)** | -9.67 (28.76) | 0.85 | -0.7 |
| self-improvement [Selbstverbesserung] | achievement** | 18.7 (18.96)*** | 5.65 (25.82) | 0.49 | -0.71 |
| motivated [motiviert] | achievement** | 29.9 (13.61)*** | 12.9 (23.84) | 0.8 | -0.71 |
| efficient [effizient] | achievement** | 18.78 (19.15)*** | -5.48 (30.11) | 1.82 | -1.15 |
| determined [strebend] | achievement** | 12.48 (20.92)* | 2.15 (26.91) | 1.78 | -1.25 |
| pressure-to-succeed [Erfolgsdruck] | achievement** | -19.98 (18.47)*** | -4.12 (31.02) | 0.72 | -0.46 |
| drive [Eigenantrieb] | achievement** | 23.9 (16.6)*** | 7.9 (24.5) | 0.73 | -0.51 |
| competitive [wetteifernd] | achievement* | -2.23 (21.37) | 4.98 (26.53) | 1.36 | -0.92 |
| gift [Begabung] | achievement* | 29.85 (13.42)*** | 9.58 (26.44) | 0.49 | -0.72 |
| active [tatkräftig] | achievement | 23.92 (16.93)*** | 15.9 (21.69)* | 0.74 | -0.63 |
| obstinate [hartnäckig] | achievement | 10.17 (16.39)* | 10.67 (23.07) | 1.32 | -0.99 |
| rational [rational] | achievement | 11.02 (18.17)* | -17.92 (25.17)* | 1.08 | -0.9 |
| outstanding [herausragend] | achievement | 24.47 (18.84)*** | 16.3 (20.79)** | 1.6 | -1.01 |
| realistic [realistisch] | achievement | 18.88 (16.98)*** | -11.42 (27.8) | 0.66 | -0.61 |
| victory-oriented [siegesorientiert] | achievement | 7.52 (21.83) | 1.8 (27.01) | 0.86 | -0.58 |
| persistent [beharrlich] | achievement | 15.75 (16.28)*** | 2.27 (21.16) | 0.97 | -0.62 |
| well-calculated [wohlkalkuliert] | achievement | 9.33 (15.08)* | -15.38 (23.71) | 1.56 | -1.06 |
| self-critical [selbstkritisch] | achievement | 20.22 (20.01)*** | 6.62 (26.16) | 0.03 | -0.21 |
| competitive2 [kompetitiv] | achievement | 1.6 (17.18) | -2.9 (24.39) | 0.85 | -0.5 |
| conventional [angepasst] | affiliation*** | -13.98 (17.11)** | -18.75 (24.38)** | -2.3 | -0.22 |
| ingratiating [einschmeichelnd] | affiliation*** | -13.52 (19.67)* | -13.05 (23.12) | -2.02 | -0.65 |
| entertaining [unterhaltsam] | affiliation*** | 27.58 (12.81)*** | 11.58 (25.31) | -1.38 | -0.32 |
| peer-pressure [Gruppendruck] | affiliation** | -29 (18.52)*** | -9.2 (26.53) | -1.22 | -0.19 |
| excluded [ausgeschlossen] | affiliation* | -23.35 (29.77)** | -2 (29.47) | -2.23 | 0.46 |
| attached [anhänglich] | affiliation* | -10.67 (15.73)* | -10.67 (22.01) | -2.73 | 0.07 |
| sycophantic [anbiedernd] | affiliation* | -26.32 (17.36)*** | -12.05 (28.09) | -1.25 | 0.15 |
| assimilated [assimiliert] | affiliation* | -10.65 (17.34) | -14.17 (21.66) | -1.46 | 0.02 |
| popularity [Beliebtheit] | affiliation* | 23.25 (12.55)*** | 7.48 (24.5) | -1.13 | -0.35 |
| conformist [konformistisch] | affiliation | -16.92 (17.57)*** | -19.43 (20.65)*** | -1.55 | -0.31 |
| obliging [gefällig] | affiliation | -4.55 (18.82) | -11.12 (22.22) | -1.81 | -0.39 |
| inexperienced [unbedarft] | affiliation | 0.7 (20.17) | -10.27 (23.38) | -1.83 | -0.32 |
| affable [umgänglich] | affiliation | 27.62 (13.98)*** | -1.25 (29.9) | -2.53 | -1.02 |
| obedient [folgsam] | affiliation | -7.6 (19.21) | -14.55 (22.26) | -1.88 | 0.21 |
| talkative [gesprächig] | affiliation | 20.88 (17.11)*** | 4.8 (27.71) | -1.24 | -0.38 |
| popular [beliebt] | affiliation | 26.65 (13.82)*** | 6.88 (27.15) | -2.05 | -0.85 |
| communicative [mitteilsam] | affiliation | 15.03 (14.45)*** | 2.45 (23.07) | -1.39 | -0.37 |
| gregarious [gesellig] | affiliation | 30.5 (13.49)*** | 6.58 (25.56) | -2.7 | -1.01 |
| sociable [kontaktfreudig] | affiliation | 30.7 (14.87)*** | 9.25 (27.91) | -2.35 | -1.06 |
| subservient [linientreu] | affiliation | -4.08 (22.86) | -10 (28.38) | -0.53 | -0.47 |
| livid [fuchsteufelswild] | anger*** | -23.98 (17.09)*** | 12.08 (32.67) | 0.86 | 3.93 |
| irritable [reizbar] | anger*** | -24.1 (15.7)*** | 8.98 (25.9) | 0.73 | 3.61 |
| excitable [erregbar] | anger*** | -2.3 (20.57) | 18.47 (24.98)** | 0.71 | 3.34 |
| quick-tempered [hitzig] | anger*** | -10.65 (17.89) | 16.75 (23.58)* | 0.88 | 3.12 |
| impulsive [impulsiv] | anger*** | -2.2 (18.79) | 22.85 (20.46)*** | 0.78 | 3.18 |
| short-tempered [aufbrausend] | anger*** | -20.95 (18.51)*** | 14.35 (27.79) | 0.43 | 2.01 |
| hateful [hasserfüllt] | anger*** | -42.33 (10.78)*** | 8.12 (36.86) | 0.5 | 1.99 |
| aggressive [aggressiv] | anger*** | -31 (18.46)*** | 8.55 (34.89) | 0.87 | 3.4 |
| argumentative [streitlustig] | anger*** | -21.62 (22.96)*** | 7.05 (30.6) | 1.04 | 3.38 |
| upset [aufgebracht] | anger*** | -14.15 (15.68)*** | 9.42 (26.02) | 0.48 | 1.77 |
| outrage [Empörung] | anger*** | -10.62 (20.64) | 10.48 (28.98) | 0.4 | 1.89 |
| resentment [Groll] | anger*** | -30.25 (14.01)*** | 2 (31.37) | 0.31 | 2 |
| raging [rasend] | anger*** | -24.68 (17.23)*** | 13.17 (31.69) | 0.41 | 1.94 |
| angry [wütend] | anger*** | -24.7 (18.67)*** | 12.17 (30.5) | 0.37 | 1.99 |
| ill-tempered [übellaunig] | anger*** | -35.08 (11.31)*** | -0.1 (29.79) | 0.33 | 1.92 |
| grouchy [grießgrämig] | anger*** | -29.75 (13.82)*** | -9.17 (25.29) | 0.33 | 1.76 |
| annoyed [genervt] | anger*** | -27.38 (15.11)*** | 2.73 (28.29) | 0.48 | 1.67 |
| rebellious [rebellisch] | anger*** | 7.95 (18.14) | 20.1 (20.35)*** | 0.78 | 2.73 |
| offended [beleidigt] | anger*** | -25.75 (13.47)*** | -5.38 (25.56) | 0.19 | 2.81 |
| indignant [ungehalten] | anger** | -15 (17.15)*** | 10 (22.86) | 0.33 | 1.59 |
| tempestuous [stürmisch] | anger** | 1.52 (19.97) | 18.15 (25.77)* | 0.34 | 1.5 |
| spirited [temperamentvoll] | anger* | 9.05 (17.78) | 20.45 (19.06)*** | 0.83 | 2.2 |
| resistance [Widerstand] | anger* | 8.12 (21.37) | 13.73 (20.25)* | 0.36 | 1.52 |
| hostile [feindselig] | anger* | -36.8 (13.34)*** | 3.3 (29.64) | 0.7 | 2.94 |
| booming [aufmüpfig] | anger | -10.92 (15.33)* | 7.33 (22.65) | 0.43 | 1.62 |
| defiance [Trotz] | anger | -17.02 (17.27)*** | 3.7 (26.81) | 0.19 | 1.42 |
| willing-to-fight [kampfbereit] | anger | 7.67 (23.77) | 17.08 (23.78)* | 1.05 | 1.79 |
| stubborn [stur] | anger | -17.83 (20.7)*** | 0.3 (25.88) | 1.29 | 1.9 |
| destructive [zerstörerisch] | anger | -34.25 (18.44)*** | 12 (32.11) | 0.5 | 1.56 |
| rebellion [Auflehnung] | anger | 2.9 (19.85) | 12.23 (16.59)** | 0.47 | 1.37 |
| warm-hearted [warmherzig] | care*** | 37.47 (10.95)*** | 7.2 (28.53) | -2.38 | -1.5 |
| affectionate [liebevoll] | care*** | 36.75 (11.83)*** | 14.8 (29.07) | -2.4 | -1.47 |
| helpful [hilfsbereit] | care*** | 37.05 (11.23)*** | 4.27 (30.44) | -2.52 | -1.45 |
| merciful [barmherzig] | care*** | 29.7 (15.45)*** | -1.4 (30.76) | -2.17 | -1.41 |
| cordial [herzlich] | care*** | 33.95 (17.02)*** | 13.92 (27.63) | -2.38 | -1.4 |
| paternal [väterlich] | care*** | 19.75 (18.27)*** | -2.8 (29.76) | -1.76 | -1.29 |
| soft-hearted [weichherzig] | care*** | 16.75 (15.21)*** | -3.05 (27.44) | -2.42 | -1.22 |
| kind [lieb] | care*** | 34.2 (14.9)*** | 5.23 (30.55) | -2.34 | -1.35 |
| self-sacrificing [aufopfernd] | care*** | 11.77 (22.95) | 5 (24.31) | -2.18 | -1.24 |
| selfless [selbstlos] | care*** | 18.5 (21.6)*** | 2.98 (27.71) | -1.17 | -0.66 |
| consoling [tröstend] | care*** | 26.3 (15.6)*** | 2.65 (28.98) | -1.3 | -0.77 |
| magnanimous [großherzig] | care*** | 34.28 (13.63)*** | 6.3 (29.59) | -1.11 | -0.79 |
| motherly [mütterlich] | care*** | 24.22 (18.81)*** | -0.75 (29.53) | -1.2 | -0.68 |
| caring [fürsorglich] | care*** | 31.3 (13)*** | 0.88 (28.84) | -1.3 | -0.7 |
| sympathetic2 [mitfühlend] | care*** | 29.55 (12.65)*** | 10.17 (28.43) | -1.25 | -0.76 |
| sensitive [feinfühlig] | care*** | 29.72 (11.55)*** | 2.33 (26.72) | -1.18 | -0.52 |
| protective [beschützend] | care*** | 30.22 (12.28)*** | 7.25 (26.58) | -0.93 | -0.43 |
| supportive [unterstützend] | care*** | 31.17 (15.31)*** | 2.73 (31.2) | -1.08 | -0.73 |
| sympathetic [anteilnehmend] | care*** | 30.28 (12.49)*** | -5.35 (27.84) | -1.26 | -0.74 |
| prudent [umsichtig] | care** | 23.7 (13.37)*** | -3.62 (28.1) | -1.51 | -0.92 |
| kind-hearted [gutherzig] | care** | 29.44 (12.37)*** | 3.54 (26.5) | -1.28 | -0.69 |
| social [sozial] | care** | 29.5 (14.58)*** | 4.05 (29.81) | -2.5 | -1.29 |
| understanding [nachsichtig] | care** | 18.58 (12.86)*** | -2.5 (25.75) | -2.49 | -1.16 |
| unselfish [uneigennützig] | care** | 19.95 (21.47)*** | -6.33 (30.29) | -1.23 | -0.65 |
| cautious [behutsam] | care* | 19.2 (15.3)*** | -8.25 (25.09) | -2.14 | -0.59 |
| solidarity [Solidarität] | care* | 31.3 (14.16)*** | 4.23 (31.09) | -1.21 | -0.56 |
| pampering [verhätschelnd] | care | -19.05 (18.44)*** | -11.92 (23.36) | -1.89 | -0.44 |
| concerned [besorgt] | care | -8.4 (17.03) | -3.48 (26.42) | -2.1 | -0.29 |
| friendly [freundlich] | care | 35.17 (11.01)*** | 2.02 (29.92) | -2.5 | -1.18 |
| likeable [sympathisch] | care | 39.28 (11.79)*** | 11.05 (29.38) | -2.21 | -1.24 |
| comradely [kameradschaftlich] | care | 23.3 (22.75)*** | 2.27 (28.06) | -2.44 | -1.2 |
| generous [großzügig] | care | 30.45 (13.25)*** | 9.6 (28.29) | -0.93 | -0.64 |
| nice [nett] | care | 26.25 (15.1)*** | -2.25 (30.69) | -1.26 | -0.68 |
| frank [offenherzig] | care | 28.78 (13.51)*** | 8.92 (24.59) | -1.34 | -0.65 |
| consumerist [konsumorientiert] | consumption*** | -15.17 (19.74)** | -14.38 (27.32) | 2.23 | -1.94 |
| shoppin-mood [Kauflaune] | consumption*** | -4.25 (19.21) | 2.5 (25.89) | 1.25 | -1.07 |
| desire to buy [Kauflust] | consumption*** | -5.58 (20.35) | 1.02 (25.92) | 1.17 | -1.08 |
| passion-for-collecting [Sammelleidenschaft] | consumption*** | -0.88 (19.42) | -7.25 (27.07) | 0.93 | -0.92 |
| covetousness [Besitzdrang] | consumption*** | -12.38 (18.68)* | 0.65 (26.86) | 1.11 | -0.92 |
| pleasure-seeking [genusssüchtig] | consumption*** | -12.77 (20.25)* | 12.33 (22.37) | 1.02 | -0.82 |
| materialistic [materialistisch] | consumption*** | -16.92 (22.84)** | -7.65 (28.83) | 1.09 | -0.9 |
| materialistic2 [besitzorientiert] | consumption*** | -15.83 (19.72)** | -11.12 (25.97) | 2.39 | -1.6 |
| pride-of-ownership [Besitzerstolz] | consumption*** | -0.38 (17.34) | 6.23 (22.95) | 1.23 | -0.93 |
| acquisitive [habsüchtig] | consumption*** | -29.52 (14.85)*** | 0.65 (27.42) | 1.23 | -0.93 |
| commercialized [kommerzorieniert] | consumption*** | -20.7 (20.11)*** | -6.4 (27.73) | 2.13 | -1.58 |
| avaricious2 [raffgierig] | consumption** | -34.45 (13.73)*** | 4.98 (29.8) | 2.06 | -1.33 |
| acquiring [anschaffend] | consumption** | -8.3 (18.7) | -11.65 (21.35) | 0.92 | -0.73 |
| appetite [Appetit] | consumption** | 16.7 (13.9)*** | 10.17 (29.23) | 1.02 | -0.88 |
| hoarding [hamsternd] | consumption** | -13.88 (18.76)** | -12.83 (23.63) | 0.83 | -0.72 |
| collecting-mania [Sammelwut] | consumption** | -20.07 (21.45)*** | -8.95 (28.7) | 1.08 | -0.56 |
| amassing [anhäufend] | consumption** | -13.17 (17.46)** | -11.23 (22.16) | 0.98 | -0.65 |
| accumulating [ansammelnd] | consumption** | -8.27 (15.81) | -15.48 (21.07)* | 0.78 | -0.58 |
| acquisitiveness [Besitzgier] | consumption* | -28.07 (16.6)*** | 2.35 (29.56) | 1.18 | -0.73 |
| to-pocket [einheimsen] | consumption* | -16.02 (19.21)** | -12.95 (22.95) | 0.98 | -0.85 |
| bon-vivant [genießerisch] | consumption* | 23.12 (15.28)*** | 10.73 (27.89) | 1.01 | -0.78 |
| ravenous [heißhungrig] | consumption* | -3.12 (19.8) | 21.4 (20.46)*** | 1.12 | -0.8 |
| sensuality [Sinnesfreude] | consumption* | 27.08 (15.5)*** | 19.97 (25.58)** | 0.78 | -0.93 |
| avaricious [geldgeil] | consumption* | -29.77 (19.57)*** | 2.17 (29.39) | 2.06 | -1.37 |
| opulence [Üppigkeit] | consumption* | 0.2 (17.22) | 0.62 (24.47) | 1.23 | -0.84 |
| abundance [Überfluss] | consumption* | -7.4 (25.17) | -0.35 (27.03) | 1.09 | -0.84 |
| yearning [Verlangen] | consumption* | 2.7 (19.44) | 24.55 (17.93)*** | 1.14 | -0.84 |
| temptation [Versuchung] | consumption | 0.15 (17.76) | 17.58 (21.44)** | 1.14 | -0.78 |
| to-keep [behalten] | consumption | 1.23 (14.87) | -17.52 (21.52)** | 0.74 | -0.56 |
| grasping [habgierig] | consumption | -31.82 (15.48)*** | -2.5 (29.69) | 2.04 | -1.07 |
| cravings [Gelüste] | consumption | 3.95 (18.44) | 23.9 (18.17)*** | 1.05 | -0.66 |
| to-store [aufbewahren] | consumption | 6.77 (9.92)** | -14.3 (21.65)* | 0.5 | -0.51 |
| desire [Begierde] | consumption | 2.05 (21.21) | 23.75 (19.9)*** | 1.19 | -0.68 |
| possession [Besitz] | consumption | 3.25 (15.11) | -1.23 (25.24) | 1.16 | -0.7 |
| accumulate [scheffeln] | consumption | -18.68 (20.04)*** | -5.38 (24.96) | 1.01 | -0.71 |
| rummaging [stöbernd] | consumption | 6.2 (16.91) | -10.85 (26.51) | 0.74 | -0.93 |
| excessiveness [Maßlosigkeit] | consumption | -22.8 (23.72)*** | 7.5 (31.16) | 1.07 | -0.48 |
| gluttonous [gefräßig] | consumption | -26.77 (16.68)*** | 2.38 (28.05) | 1.18 | -0.57 |
| addicted [süchtig] | consumption | -26.35 (16.59)*** | 6.67 (30.84) | 1 | -0.59 |
| venal [käuflich] | consumption | -26.55 (19.2)*** | -13.52 (23.6) | 1.26 | -1.06 |
| profit-maximizing [gewinnmaximierend] | consumption | -4.1 (28.35) | -6.2 (30.34) | 2.12 | -1.4 |
| wanting [wollend] | consumption | 9.73 (16.31)* | 6.27 (23.14) | 1.05 | -1.27 |
| greedy [gierig] | consumption | -33.33 (14.73)*** | 7.33 (26.13) | 2.18 | -1.18 |
| lack of restraint [Hemmungslosigkeit] | consumption | -10.17 (22.89) | 22.53 (25.7)*** | 0.98 | -0.12 |
| eager [vorfreudig] | consumption | 29.8 (14.41)*** | 20.72 (23.84)*** | 0.73 | -0.84 |
| impatient [ungeduldig] | consumption | -21.82 (15.06)*** | 0.1 (25.19) | 0.86 | 0.02 |
| panic-stricken [panisch] | fear*** | -31.95 (15.68)*** | 10.6 (35.96) | -1.61 | 2.54 |
| apprehensive [ängstlich] | fear*** | -27.12 (16.11)*** | 0.05 (27.16) | -1.63 | 2.38 |
| frightened [verängstigt] | fear*** | -27.62 (14.09)*** | -0.4 (27.64) | -1.65 | 2.21 |
| timid [furchtsam] | fear*** | -21.2 (15.18)*** | -4.35 (23.03) | -1.75 | 2.29 |
| careful [vorsichtig] | fear*** | 2.3 (16.04) | -13.92 (25.13) | -1.54 | 1.77 |
| risk-averse [risikoscheu] | fear*** | -11.7 (12.8)*** | -17.05 (20.83)** | -1.32 | 1.84 |
| afraid [bange] | fear*** | -20.05 (14.94)*** | -8.17 (23.8) | -0.87 | 1.25 |
| distraught [verstört] | fear*** | -27.6 (13.4)*** | 0.62 (27.85) | -0.77 | 1.34 |
| nervous [nervös] | fear*** | -21.73 (15.98)*** | 7.02 (26.36) | -0.78 | 1.28 |
| overcautious [übervorsichtig] | fear*** | -19.07 (15)*** | -8.5 (22.13) | -0.83 | 0.93 |
| worried [beunruhigt] | fear*** | -17.9 (13.26)*** | -0.85 (23.28) | -0.66 | 1.26 |
| subdued [kleinlaut] | fear*** | -17.95 (13.98)*** | -22.32 (19.58)*** | -1.58 | 1.82 |
| alarmed [alarmiert] | fear** | -9.73 (14.28)* | 10.62 (25.83) | -0.58 | 1.13 |
| bemused [irritiert] | fear** | -11.92 (11.82)*** | -3.98 (26.36) | -0.83 | 1.18 |
| desperate [verzweifelt] | fear** | -33.3 (17.12)*** | 5.88 (30.28) | -0.92 | 1.07 |
| vigilant [wachsam] | fear* | 16.53 (12.79)*** | 3.33 (21.53) | -0.82 | 0.96 |
| self-protective [selbstschützend] | fear* | 15.62 (15.94)*** | -1.88 (27.33) | -0.48 | 1.01 |
| tense [angespannt] | fear | -15.6 (17.15)*** | 1.48 (26.24) | -0.78 | 2.19 |
| defensive [defensiv] | fear | -5.98 (18.39) | -18.55 (23.14)** | -1.52 | 1.61 |
| overwhelmed [überfordert] | fear | -28.07 (15.79)*** | -2.75 (29.91) | -0.44 | 0.95 |
| disconcerted [aufgewühlt] | fear | -16.85 (15)*** | 8.95 (23.68) | -0.5 | 1.37 |
| thin-skinned [dünnhäutig] | fear | -19.6 (13.77)*** | -9.92 (25.43) | -0.67 | 1.31 |
| obsequious [unterwürfig] | fear | -27.15 (17.19)*** | -9.15 (25.81) | -0.9 | 0.75 |
| fear of failure [Versagensangst] | fear | -33.98 (14.65)*** | 8.25 (30.65) | -0.34 | 0.65 |
| authoritarian [autoritär] | power*** | -16.88 (21.31)** | 7.9 (27.8) | 2.09 | 0.43 |
| officious [tonangebend] | power*** | -8.65 (18.7) | 9.67 (22.16) | 2.03 | 0.52 |
| mighty [mächtig] | power*** | -6.45 (20.87) | 17.47 (20.01)*** | 1.99 | 0.48 |
| influential [einflussreich] | power*** | 11.27 (16.6)** | 6.33 (21.64) | 1.74 | 0.09 |
| dominant [dominant] | power*** | -12.33 (20.44) | 16.38 (23.66)* | 1.81 | 0.47 |
| firm [bestimmt] | power*** | -9.98 (16.68) | 8.55 (22.39) | 1.92 | 0.57 |
| arrogant [arrogant] | power*** | -34.1 (14.01)*** | -1.9 (31.51) | 1.82 | 0.56 |
| manipulative [manipulativ] | power*** | -35.08 (11.78)*** | -1.15 (31.65) | 1.21 | 0.43 |
| despotic [despotisch] | power*** | -27.9 (19.84)*** | -0.62 (30.6) | 1.67 | 0.6 |
| conceited [eingebildet] | power*** | -33.65 (13.38)*** | -8.15 (30.46) | 1.47 | -0.08 |
| imperious [herrisch] | power*** | -33.65 (13.49)*** | 6.12 (29.47) | 1.7 | 1.04 |
| sly [durchtrieben] | power*** | -22.62 (24.31)*** | 8.23 (30.44) | 1.75 | 0.05 |
| self-centred [egozentrisch] | power*** | -22.07 (18.82)*** | 2.88 (29.57) | 1.74 | 0.52 |
| self-important [wichtigtuerisch] | power*** | -31.48 (14.48)*** | -4.1 (28.07) | 1.17 | 0.11 |
| tyrannical [tyrannisch] | power** | -43.45 (9.79)*** | 5.15 (35.43) | 1.79 | 1.27 |
| condescending [herablassend] | power** | -35.95 (17.67)*** | -1.3 (32.62) | 1.54 | 1.31 |
| stifling [bevormunden] | power* | -26.9 (14.51)*** | -6.88 (25.14) | 0.58 | 0.31 |
| important [gewichtig] | power* | 3.25 (11.06) | -7.33 (23.66) | 0.83 | 0.18 |
| ruthless [rücksichtslos] | power* | -31.93 (17.28)*** | 1.33 (31.26) | 0.99 | 0.59 |
| significant [bedeutend] | power* | 21.55 (14.39)*** | 6.08 (22.16) | 0.51 | -0.11 |
| self-seeking [selbstsüchtig] | power | -33.45 (14.65)*** | 0.2 (29.29) | 0.99 | -0.11 |
| complacent [selbstgefällig] | power | -26.35 (16.92)*** | -8.38 (25.2) | 1.51 | -0.24 |
| selfwilled [eigenwillig] | power | 3 (15.42) | 5.95 (21.52) | 1.4 | 0.13 |
| vain [eitel] | power | -24.02 (19.01)*** | -6.75 (23.66) | 1.64 | -0.35 |
| boastful [prahlerisch] | power | -31.88 (13.56)*** | -1.7 (28.09) | 1.16 | -0.43 |
| paternalistic [paternalistisch] | power | -12.85 (17.08)** | -8.73 (22.96) | 0.06 | -0.03 |
| assertive [durchsetzungsfähig] | power | 21.78 (14.3)*** | 7.55 (22.56) | 1.84 | -0.17 |
| contemptuous [geringschätzig] | power | -30.6 (14.49)*** | -6.25 (27.18) | 0.79 | 1.35 |
| strong [stark] | power | 29.17 (13.28)*** | 17.35 (24.74)* | 0.99 | -0.08 |
| convincing [überzeugend] | power | 21.45 (16.76)*** | 5.85 (23.35) | 0.85 | -0.1 |
| disparaging [verächtlich] | power | -30.68 (14.57)*** | 0.33 (29.1) | 1.36 | 1.64 |
