# Supplementary material for: Navigating Motivation: A Semantic and Subjective Atlas of 7 Motives
Source: Front Psychol. 2021 Jan 27;11:568064. doi: 10.3389/fpsyg.2020.568064 (PMC7874174; doi:10.3389/fpsyg.2020.568064)
Supplement: Supplementary file 2 [file Image_1.pdf]

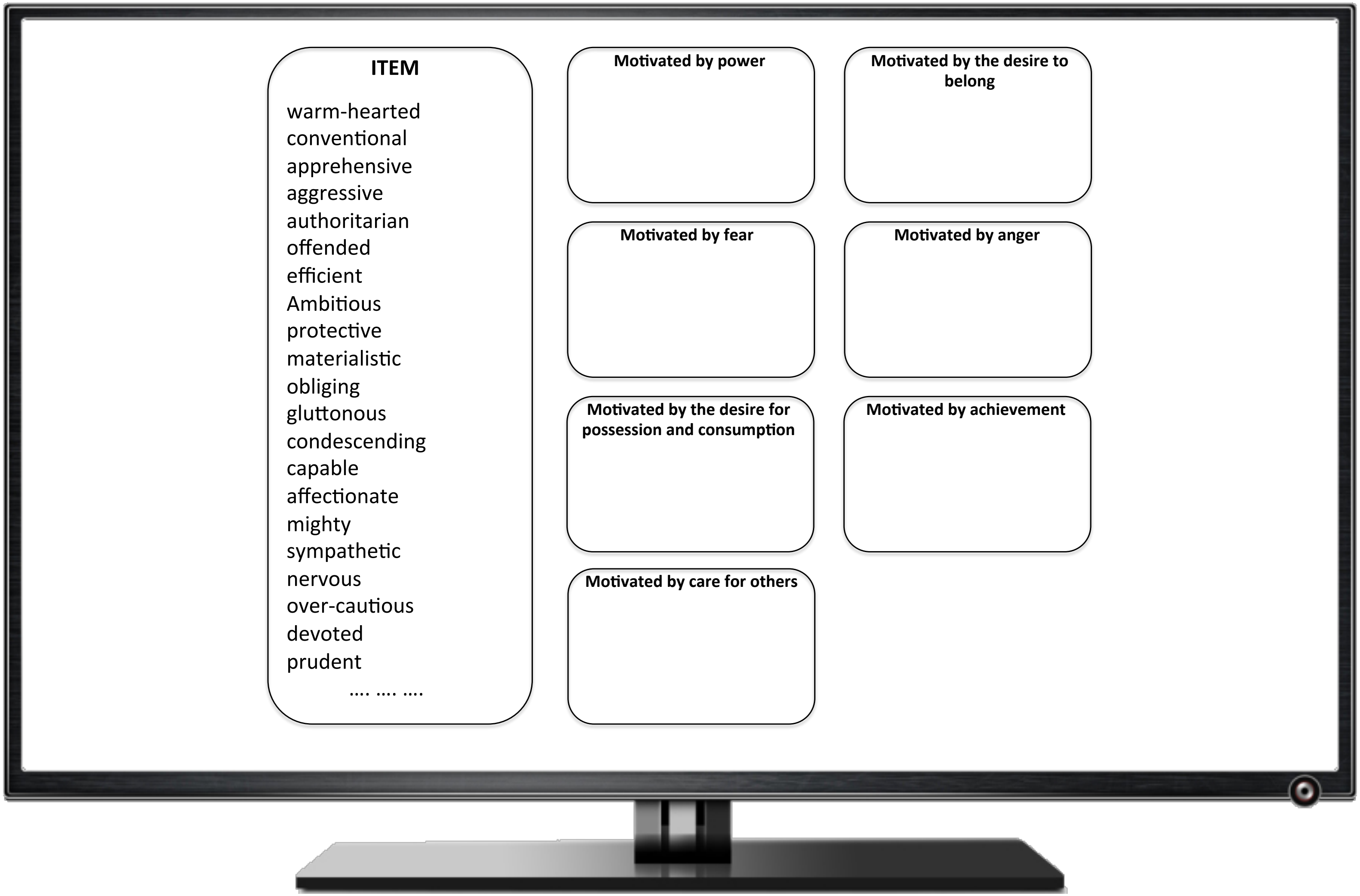


**Supplementary figure 1:** Example screen display during the semantic categorization task. Participants were shown a list of either 174 or 304 words (random order) on the left and were asked to drag and drop each of the words into one of the seven boxes on the right. Each of the boxes represented one of the seven postulated motives. There were no further instructions regarding time limit or number of words that should be sorted into the boxes.
